# Differential Mechanisms of Potato Yield Loss Induced by High Day and Night Temperatures During Tuber Initiation and Bulking: Photosynthesis and Tuber Growth

**DOI:** 10.3389/fpls.2019.00300

**Published:** 2019-03-14

**Authors:** Yean-Uk Kim, Byun-Woo Lee

**Affiliations:** ^1^Department of Plant Science, College of Agriculture and Life Sciences, Seoul National University, Seoul, South Korea; ^2^Research Institute of Agriculture and Life Sciences, Seoul National University, Seoul, South Korea

**Keywords:** potato, high night temperature, high day temperature, growth stage, photosynthesis, tuber growth

## Abstract

The tuber yield of potatoes is vulnerable to high temperature and is challenged by the asymmetric increase in day and night temperatures. This study aimed to evaluate photosynthesis, biomass growth, tuber mass distribution, and dry tuber yield in early harvested potatoes that were field-grown under high day and night temperature conditions during different growth stages. Potatoes were exposed to ambient (control), high night temperature (HNT; 19:00–7:00), high day temperature (HDT; 7:00–19:00), and high day/night temperature (HDNT; all day) for 14 days during tuber initiation (TI) or tuber bulking (TB) using portable, temperature-controlled plastic houses that were controlled to increase the temperature by 4.0°C. During TI, HNT delayed tuber development, thus altering tuber mass distribution by reducing the yield proportion of large tubers of >100 g (-53.7%) and lowering early harvest index (-16.1%), causing a significant yield loss (-17.2%) without interfering with photosynthesis. In contrast, HDT decreased early tuber yield (-18.1%) by reducing photosynthetic sources, which was probably attributed to decreased photosynthetic efficiency through a feedback inhibition. However, HDT altered neither tuber mass distribution nor early harvest index. HDNT during TI exhibited all the aforementioned effects of HNT and HDT (i.e., cumulative effects): reduced yield proportion of large tubers (-46.7%), decreased early harvest index (-23.7%), and reduced photosynthetic rate; thus, HDNT caused the highest yield loss (-30.3%). During TB, when the tubers were fully developed, the thermal effects decreased because most of the effects were either directly or indirectly linked to tuber development. These results provide comprehensive insight to the differential mechanisms of potato yield loss under high day and night temperatures and show that further field experiments should be conducted to cope with the threat of global warming on potato production.

## Introduction

The global daily mean temperature is expected to increase by 1.0–3.7°C by the end of the 21st century (IPCC, 2013). The increase in minimum temperature during the night has been greater than the increase in maximum temperature during the day, reducing diurnal temperature range (DTR) on a global scale (Easterling et al., 1997; Harris et al., 2014). Reduced DTR has been shown to affect crop growth and development (Benoit et al., 1986; Yin et al., 1996; Bahuguna and Jagadish, 2015). It is also reported that crop growth and yield are adversely affected by high day temperature (Matsui et al., 1997; Elía et al., 2018) or high night temperature (Prasad and Djanaguiraman, 2011; García et al., 2015).

Potatoes are one of the most important food crops worldwide^1^, and this crop is highly vulnerable to high temperature (Levy and Veilleux, 2007). The adverse effects of increased mean daily temperature on potato yield has been recently evaluated with greenhouse or growth chamber experiments (Kim et al., 2017; Rykaczewska, 2017) and modeling studies (Fleisher et al., 2017). The general responses of different processes in potato plant to temperature are well documented, as outlined below. Tuber yield and dry matter partitioning have an optimum temperature of approximately 20°C (Bodlaender, 1963; Marinus and Bodlaender, 1975; Timlin et al., 2006). However, tuber development has lower optimum temperatures: tuber induction is optimal at 15°C, initiation at 22°C, and setting at 15°C (Struik, 2007). Therefore, the critical period for potato yield and tuber size is when the heat-sensitive sink (i.e., tuber) develops (Struik, 2007). Leaf photosynthesis is known to be less sensitive to high temperature than tuber development, with an optimum temperature of approximately 24°C (Ku et al., 1977; Leach et al., 1982; Timlin et al., 2006). It has been suggested that reduction of photosynthesis under high temperature is mainly caused by decreased efficiency of photosystem II rather than decreased stomatal conductance (Dwelle et al., 1981; Prange et al., 1990).

Although night temperature, compared to day temperature, is believed to have profound effects on potato growth and yield (Gregory, 1956; Sale, 1979; Levy and Veilleux, 2007), the independent effects of high day and night temperatures and their interaction on potato growth were examined only with pot experiments (Benoit et al., 1986), which have several limitations. Belowground organs, including the tubers, stolons, and roots, may be exposed to unnatural environments, such as increased soil temperature caused by light striking the side of pots (Passioura, 2006) and limited space for belowground growth. Meanwhile, several field experiments in other crops, such as rice and wheat, were conducted to examine stress induced by high day and night temperatures (Shah et al., 2014; Fang et al., 2015). Similar field experiments in potatoes are required to cope with the asymmetric increase in day and night temperatures.

Potatoes are often harvested before maturation for specific purposes. Early potatoes are preferred for their economic benefit of high market prices in the early part of the harvesting season (Jenkins and Gillison, 1995). In Poland, the demand for early harvested potato tubers increases in mid-May, when the tubers from the previous year’s harvest are already tasteless (Jablońska-Ceglarek and Wadas, 2005). In South Korea, growing-season length of spring potato, which occupies more than 60% of annual potato production, is limited by frost at the early spring and by rainy spell at the late growth. Thus, potatoes are harvested within 100 days after planting. In the present study, a field experiment was conducted to investigate the photosynthesis, biomass growth, tuber mass distribution, and yield responses of early harvested potato to high day and night temperatures during different growth stages: tuber initiation (TI) and tuber bulking (TB).

## Materials and Methods

### Crop Management

The field experiment was conducted at the experimental farm of Seoul National University in Suwon, South Korea (37.27 °N, 126.99 °E) during the warm season of 2018. The general soil characteristics are presented in Table 1. N-P-K fertilizer of 100–100–120 kg ha^-1^ was uniformly applied before planting. On May 1, 2018, sixteen pre-sprouted seed potato pieces (30–50 g) per plot were planted in 0.7-m-spaced rows, with a plant spacing of 0.25 m. The early maturing Superior variety, which is the leading potato variety in South Korea, was chosen for the experiment. Weeds, pests, and pathogens were controlled with recommended chemical treatments and with hand-weeding during mid to late growth. Plots covered with plastic houses were manually irrigated after rainfall, in accordance with rainfall data from a weather station located within 500 m from the field. Total rainfall from planting to harvest was 503 mm, providing sufficient water for short-growing-season potatoes (Doorenbos and Kassam, 1979).

**Table 1:** General soil characteristics of the experimental field.

Soil depth (cm)	Soil texture	Sand (%)	Silt (%)	Clay (%)	Bulk density (g cm^-3^)	pH^a^	Total organic carbon (%)	Total nitrogen (%)	CEC (cmol^(+)^ kg^-1^)
0–15	Sandy loam	62.2	20.4	17.3	1.17	6.90	2.02	0.19	14.1
15–30	Sandy loam	59.9	22.5	17.7	1.40	6.83	1.57	0.15	13.5
30–45	Sandy loam	62.7	21.3	16.0	1.50	5.93	0.69	0.07	11.7
45–60	Loam	42.4	31.3	26.3	1.38	5.27	1.00	0.11	14.7
60–75	Loam	45.6	33.4	21.0	1.52	4.87	0.79	0.08	12.8
75–90	Loam	42.7	37.3	20.0	1.67	5.23	0.38	0.06	13.5


*^a^pH was determined in a 1:5 soil-to-water suspension*.

### Experimental Design and Temperature Control

The potatoes were subjected to four different thermal regimes, namely ambient temperature (control), high night temperature (HNT; 19:00–7:00), high day temperature (HDT; 7:00–19:00), and HDNT (all day) during two different growth stages: TI and TB. The onset of TI (TIO) was destructively checked during 12–18 days after emergence (DAE) from the field right next to the experimental plots. Five to ten plants were sampled every 2 days, and TIO was defined as the date when more than 50% plants had at least one stolon tip starting to swell (i.e., stolon tip more than twice in size the stolon diameter). At 31 DAE, 12 plants were sampled to confirm that tubers had started to bulk, and that every plant had more than 50 g of fresh tuber mass at the time. The thermal treatments were maintained for 14 days: from 19:00 on the first day of warming (TI: 18 DAE; TB: 33 DAE) to 19:00 on the final day of warming (TI: 32 DAE; TB: 47 DAE). Temperature was increased to the target temperature of ambient + 4.0°C using a portable temperature-controlled plastic house equipped with temperature and relative humidity sensors (SHT75; Sensirion AG, Switzerland) mounted at the canopy height and two electric fan heaters installed at both ends of the house. The target temperature approximated the projected daily average temperature increase of 2.8–5.3°C by the end of this century in South Korea under the Representative Concentration Pathways (RCP) 4.5 and 8.5 scenarios (Korea Meteorological Administration [KMA], 2012). During daytime, the sidewalls of HDT and HDNT were opened by 10–20 cm to avoid overheating, whereas those of the control and HNT were fully opened. During nighttime, the sidewalls of HNT and HDNT were closed to prevent heat loss. Radiations inside and outside one of the plastic houses were observed by two pyranometers (SP-212; Apogee Instruments, Inc., United States). The plots were laid out in a randomized complete block design in three replicates.

### Measurements

#### Tuber Yield and Related Traits

At 55 and 56 DAE, 5 plants near the temperature and relative humidity sensors (i.e., in the middle of the plastic house) were manually harvested from each plot. The samples were separated into shoots and tubers, excluding roots and stolons. Aboveground biomass was measured after oven-drying until a constant mass was reached. The tubers were manually washed with water, air-dried for approximately 1 h, and then weighed to measure the fresh mass of individual tubers. The tubers with a fresh mass of lower than 1 g were regarded as developing tuber initials (i.e., stolon tips that are not perfectly spherical), and thus were excluded from the samples. To investigate thermal effects on tuber mass distribution, the tubers were categorized into three mass classes: small (1–50 g), medium (50–100 g), and large (>100 g). Two plants per plot were selected to determine tuber dry matter content and tuber yield. The tubers from the selected plants were sliced into 1–2-cm-thick sections, oven-dried until a constant mass was reached, and then weighed. Tuber yield of the rest of the plants was calculated as the product of dry matter content and tuber fresh mass. Harvest index was determined as tuber yield over total biomass, calculated as the sum of aboveground biomass and tuber yield.

#### Gas Exchange and Chlorophyll Fluorescence

Light-saturated net CO_2_ assimilation rate (A_max_), stomatal conductance for H_2_O (G_Smax_), and leaf chlorophyll fluorescence were measured simultaneously on the terminal leaflets of the youngest, fully expanded leaves (i.e., the fourth to sixth leaf from the top of canopy) from 1 or 2 plants per plot using a portable photosynthesis system (LI-6400; LI-COR Biosciences, United States) integrated with a leaf chamber fluorometer (LI-6400-40; LI-COR Biosciences, United States). The leaves were light-adapted at a saturating irradiance of 1500 μmol PAR m^-2^ s^-1^ until gas exchange and chlorophyll fluorescence parameters were stabilized (i.e., for 10–30 min, depending on the time of day and weather condition). The leaf chamber was controlled at a flow rate of 500 μmol s^-1^, with a reference CO_2_ concentration of 400 μmol mol^-1^ and a reference relative humidity within 50 to 70%. Block temperatures were adjusted to match real-time temperature data from the temperature and relative humidity sensors in each plot. A_max_, G_Smax_, and steady-state fluorescence (F_s_) were measured after the parameters stabilized. Next, an 800-ms saturating flash (>7000 μmol m^-2^ s^-1^) was applied to determine the maximum fluorescence yield of illuminated leaves (F_m_′). Effective quantum yield of photosystem II (Φ_PSII_) was calculated as 1 – (F_s_/F_m_′). All parameters were measured at 1, 3, 10, 11, 19, 21, and 22 days after the onset of thermal treatment (DAT) for TI and at 3, 5, 11, 13, 15, and 20 DAT for TB. On each day, measurements started in the first block (i.e., replication), then in the second, and finally in the third. The average measurement times-of-day was 11:00, 13:00, and 14:50 for the first, second, and third blocks, respectively. In some cases, the values of reference CO_2_ concentration, reference relative humidity, and intercellular CO_2_ concentrations were too low owing to gas depletion in the CO_2_ cylinder, dry days, and measurement failures caused by wet leaves, respectively. In these cases, the datasets were discarded.

### Statistical Analysis

All statistical analyses were performed using SAS software version 9.4 (SAS Institute Inc., United States). For the agronomic traits, subsampled data (five plants) were averaged for each plot. Then, averages and standard errors were calculated for each thermal regime based on the three blocks. To evaluate the effects of the thermal regimes on tuber yield and related traits (aboveground biomass, total biomass, harvest index, and tuber mass distribution), analysis of variance (ANOVA; thermal regime and block as fixed factors) and least significant difference (LSD) test were performed for each growth stage (TI and TB) using PROC ANOVA procedure. The gas exchange and chlorophyll fluorescence data that had been measured on two plants per plot were first averaged for each plot. Then, data were pooled for three different phases of treatment: early (TI: 1, 3 DAT; TB: 3, 5 DAT), late (TI: 10, 11 DAT; TB: 11, 13 DAT), and subsequent (TI: 19, 21, 22 DAT; TB: 15, 20 DAT). For each phase of treatment, the effects of thermal regimes on A_max_, G_Smax_, and Φ_PSII_ were assessed by ANOVA with repeated measures over time (DAT) and LSD test using PROC MIXED procedure. Pearson’s correlations between A_max_, G_Smax_, and Φ_PSII_ were calculated for each growth stage using PROC CORR procedure. To determine associations among agronomic and photosynthetic traits and to visualize differences among thermal regimes, principal component analysis (PCA) was conducted using PROC PRINCOMP procedure.

## Results

### Climate Conditions and Temperature Control

**FIGURE 1:**
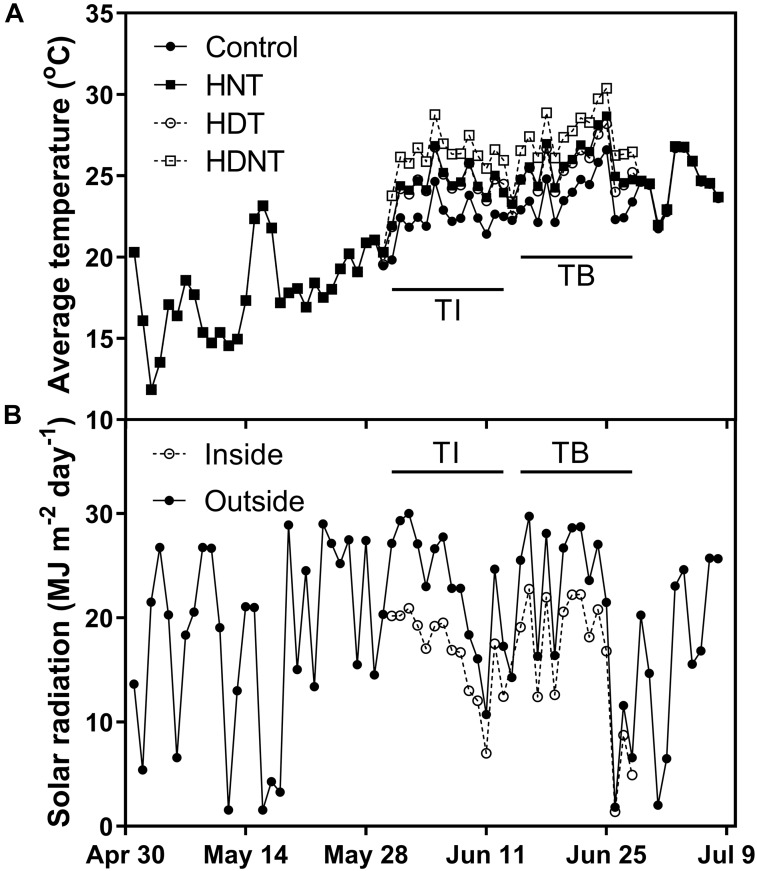
Daily average temperatures under different thermal regimes **(A)** and daily total solar radiation inside and outside of the plastic house **(B)**.

The mean daily average ambient temperature throughout the crop cycle (0–56 DAE) was 21.9°C (Figure 1A). The ambient temperature was 22.2 and 23.7°C during TI and TB, respectively. Both day and night temperatures were successfully elevated to the target temperature of ambient + 4.0°C during both TI and TB (Table 2). All thermal regimes were within 0.4°C of the target temperature difference. The mean daily total solar radiation during the growing season was 19.5 MJ m^-2^ d^-1^, and the light transmittance of the plastic houses was 74% (Figure 1B). Relative humidity was not altered by HNT, whereas HDT and HDNT decreased relative humidity by -2.5 and -2.0%p, respectively. The mean daily average relative humidity throughout the crop cycle was 71.1% for the control plots.

**Table 2:** Day and night temperatures in four thermal regimes during tuber initiation (TI) and tuber bulking (TB).

Growth stage	Thermal regime	Mean day temperature (°C)	Mean night temperature (°C)
TI	Control	26.8	17.8
	HNT	27.2 (+0.4)	21.7 (+4.0)
	HDT	30.7 (+3.9)	17.9 (+0.2)
	HDNT	30.8 (+4.0)	21.8 (+4.0)
TB	Control	27.5	20.0
	HNT	27.8 (+0.2)	24.1 (+4.1)
	HDT	31.2 (+3.7)	20.0 (+0.0)
	HDNT	31.2 (+3.7)	24.1 (+4.1)


*Mean day and night temperatures represent the mean daily day (7:00–19:00) and night (19:00–7:00) temperatures, respectively. Values in parentheses indicate temperature differences between thermal regimes and control for each growth stage*.

### Tuber Yield and Related Traits

Both high day and night temperatures during TI lowered tuber yield (Table 3). HNT, HDT, and HDNT reduced tuber yield by 17.2, 18.1, and 30.3%, respectively. Aboveground biomass was increased by 26.0 and 26.9% by high night temperatures (HNT and HDNT, respectively), but not affected by HDT. Although the effect of the thermal regimes on total biomass was not significant at *p* < 0.05, HDT tended to reduce total biomass by 14.6%. Collectively, harvest index was 16.1 and 23.7% lower in HNT and HDNT than in the control, respectively, but not significantly affected by HDT. The yield proportion of large tubers was decreased from 0.41 (control) to 0.19 and 0.22 by HNT and HDNT, respectively, but not significantly affected by HDT. The yield proportions of small and medium tubers were not affected by the thermal regimes. High temperatures during TB had no significant effect on tuber yield and related traits.

**Table 3:** Tuber yield, aboveground biomass, total biomass, harvest index, and tuber mass distribution (means ± SE) of the potatoes grown under four different thermal regimes at TI and TB.

Growth stage	Thermal regime	Tuber yield (g DM plant^-1^)	Aboveground biomass (g DM plant^-1^)	Total biomass (g DM plant^-1^)	Harvest index	Yield proportion		
						Small	Medium	Large
TI	Control	78.1 ± 4.9 a	37.9 ± 7.1 b	115.9 ± 10.7	0.68 ± 0.04 a	0.35 ± 0.08	0.24 ± 0.04	0.41 ± 0.12 a
	HNT	64.6 ± 6.8 b	47.7 ± 5.2 a	112.4 ± 7.7	0.57 ± 0.04 b	0.48 ± 0.07	0.33 ± 0.08	0.19 ± 0.05 b
	HDT	63.9 ± 6.0 b	35.1 ± 2.7 b	99.0 ± 7.7	0.65 ± 0.02 a	0.34 ± 0.10	0.26 ± 0.01	0.40 ± 0.10 a
	HDNT	54.4 ± 2.5 b	48.1 ± 2.7 a	102.5 ± 2.8	0.52 ± 0.03 c	0.49 ± 0.10	0.29 ± 0.09	0.22 ± 0.03 b
	Significance	^∗^	^∗^	ns	^∗∗∗^	ns	ns	^∗^
TB	Control	82.3 ± 2.3	43.7 ± 2.3	126.0 ± 4.2	0.66 ± 0.01	0.31 ± 0.03	0.38 ± 0.01	0.31 ± 0.04
	HNT	84.3 ± 2.4	42.6 ± 0.6	126.8 ± 2.3	0.66 ± 0.01	0.36 ± 0.04	0.37 ± 0.10	0.28 ± 0.06
	HDT	89.7 ± 13.4	46.9 ± 6.4	136.6 ± 19.7	0.65 ± 0.01	0.33 ± 0.02	0.41 ± 0.04	0.27 ± 0.04
	HDNT	83.4 ± 3.4	47.3 ± 6.2	130.7 ± 9.2	0.64 ± 0.02	0.32 ± 0.03	0.33 ± 0.04	0.35 ± 0.06
	Significance	ns	ns	ns	ns	ns	ns	ns


*Yield proportion is determined as tuber yield (FM) of each mass class over total tuber yield (FM). Small, medium, and large represent tuber mass classes of 1–50, 50–100, and >100 g, respectively. ^∗^*p* < 0.05, ^∗∗∗^*p* < 0.001, and ns, non-significant, respectively. Within each growth stage, the same letters in a column are not significantly different, as analyzed by LSD test at the 0.05 probability level*.

### Gas Exchange and Chlorophyll Fluorescence

**FIGURE 2:**
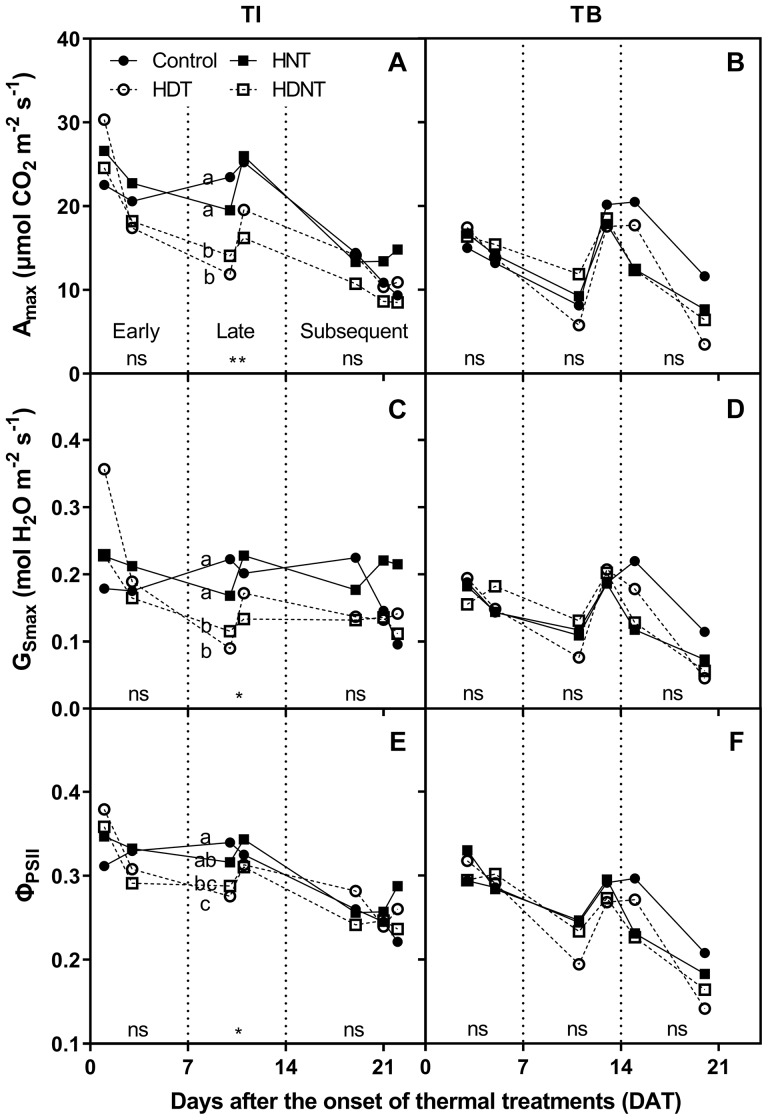
Light-saturated net CO_2_ assimilation rate (A_max_; **A,B**), stomatal conductance for H_2_O (G_Smax_; **C,D**), and effective quantum yield of photosystem II (Φ_PSII_; **E,F**) of the potatoes grown under four different thermal regimes at tuber initiation (TI; **A,C,E**) and tuber bulking (TB; **B,D,F**). The vertical dotted lines divided the *x*-axis to the early, late, and subsequent phases of treatment; ^∗^*p* < 0.05, ^∗∗^*p* < 0.01, and ns, non-significant, respectively. Different letters within each phase of treatment denote significant differences among the thermal regimes tested by LSD test at *p* < 0.05. The letters are arranged vertically in the order of control, HNT, HDNT, and HDT.

As presented in Figure 2, all thermal regimes had negligible effects on photosynthetic characteristics during the early phase of TI (i.e., 1st week after TIO). However, within the late phase of TI (i.e., 2nd week after TIO), high day temperatures (HDT and HDNT) significantly lowered A_max_ by 35.5 and 37.9%, G_Smax_ by 38.3 and 41.4%, and Φ_PSII_ by 11.4 and 10.0%, respectively, whereas HNT did not affect the characteristics. At the subsequent phase of TI (i.e., 3rd to 4th week after TIO), all photosynthetic characteristics were not significantly affected by the thermal regimes. Photosynthetic characteristics at every phase of TB were not affected by the thermal regimes.

Table 4 presents the correlations between A_max_ and the other two photosynthetic traits of potatoes grown under different thermal regimes at TI and TB. The variation in A_max_ during TI was better explained by Φ_PSII_ (*r* = 0.96^∗∗∗^) compared to by G_Smax_ (*r* = 0.78^∗∗∗^). On the other hand, the variation in A_max_ during TB, which was not affected by the thermal regimes, was highly associated with both G_Smax_ (*r* = 0.96^∗∗∗^) and Φ_PSII_ (*r* = 0.86^∗∗∗^).

**Table 4:** Pearson’s correlations between light-saturated net CO_2_ assimilation rate (A_max_) and the other photosynthetic characteristics: stomatal conductance for H_2_O (G_Smax_) and effective quantum yield of photosystem II (Φ_PSII_) of the potatoes grown under four different thermal regimes at TI and TB.

	G_Smax_	Φ_PSII_
TI	0.78^∗∗∗^	0.96^∗∗∗^
TB	0.96^∗∗∗^	0.86^∗∗∗^


*^∗∗∗^*p* < 0.001*.

### Principal Component Analysis

**FIGURE 3:**
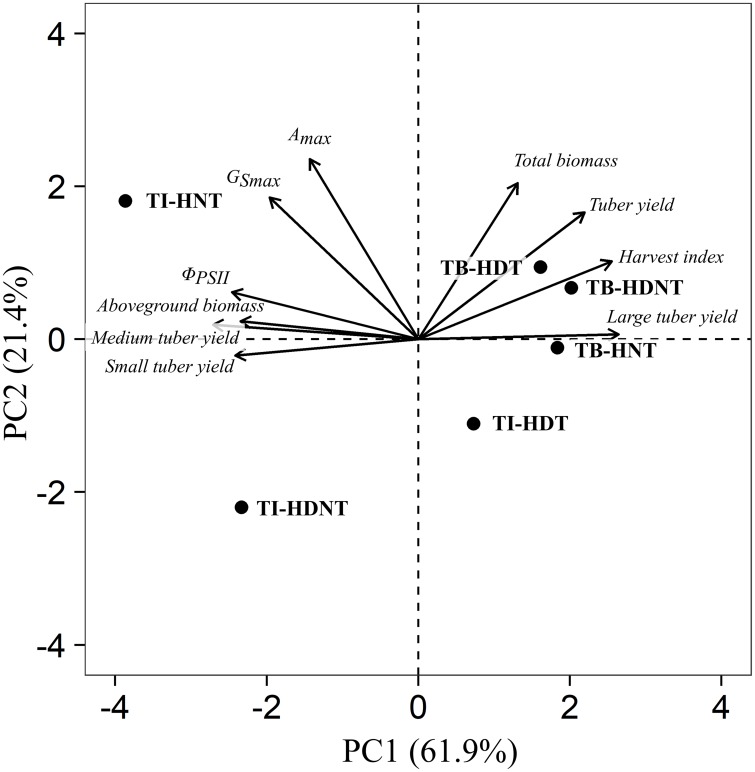
PCA bi-plot of the agronomic- and photosynthetic-traits for the potatoes grown under different thermal regimes at TI and TB. In order to remove the effect of different radiation conditions between TI and TB, the measurements of agronomic- and photosynthetic-traits were transformed to the percentage changes compared to the controls of each growth stage.

The first two principal components (PCs) could explain 83.3% of the total variation among thermal regimes at TI and TB (Figure 3). In PC1, the yield proportions for tuber mass classes (loading values for small tubers: -0.34; medium tubers: -0.38; large tubers: +0.37) and harvest index (+0.36) were the most explanatory variables. PC1 separated TI-HNT and TI-HDNT from the others. In PC2, A_max_ (0.56) was the most explanatory variable. PC2 separated TI-HDT and TI-HDNT from the others. Thermal treatments at TB, which affected neither agronomic nor photosynthetic traits, were clustered in the right side.

## Discussion

### Effect of High Night Temperature

The yield loss caused by HNT at TI was attributed to reduced harvest index and large tuber yield (Table 3 and Figure 3). High temperature, particularly HNT, is reported to delay tuber induction, prolong tuber setting, and delay the onset of rapid tuber growth, thus reducing carbohydrate partitioning in tubers at the early growth (Slater, 1968; Sale, 1979; Struik, 2007). In addition, prolonged tuber setting results in increased within-plant variations in the initiation time and bulking period of individual tubers (Struik, 2007), modifying tuber mass distribution at harvest. Collectively, HNT-induced yield loss in early harvested potatoes is attributable to delayed crop development; indicating that the effect of HNT can be different for full-growing-season potatoes, which can grow until physiological maturity (i.e., 120–180 days). Larger canopy at the early growth may extend the growing season and may increase the final tuber yield.

Photosynthesis was not significantly affected by HNT at TI (Figure 2). Although HNT may not have a direct effect on photosynthesis, decreased leaf carbohydrate caused by increased dark respiration under HNT may enhance photosynthetic capacity for the next day, as reported in other plants (Turnbull et al., 2002; Kanno et al., 2009). However, photosynthesis can also be modified by tuber sink; limited tuber sink reduces photosynthetic efficiency by accumulating carbohydrates in the leaves (Basu et al., 1999). Hence, the decrease in tuber sink strength caused by HNT at TI may offset the aforementioned effect of dark respiration, resulting in the negligible effect of HNT on A_max_.

Tuber bulking is less sensitive to high temperature than tuber formation: a 1-week period of high temperature (32/27°C; day/night) at TI reduces tuber yield, whereas that at TB has less effect on tuber yield (Struik, 2007). In the present study, the temperature was even lower, 28/24°C for HNT at TB (Table 2). Thus, HNT showed no adverse effect at TB (Table 3 and Figure 3), as was reported in a previous study regarding increased mean daily temperature (Lizana et al., 2017).

### Effect of High Day Temperature

The yield loss caused by HDT at TI (31/18°C) was attributed to reduced photosynthesis (Figure 2, 3). Net photosynthesis of potatoes is optimal at approximately 24°C and rapidly decreases as temperature increases above the optimum (Ku et al., 1977; Leach et al., 1982; Ghosh et al., 2000; Timlin et al., 2006). Photosynthesis under high temperature is known to be limited by reduced stomatal conductance, accelerated senescence, damaged photosystems, or modified source-sink relation (Prange et al., 1990; Reynolds et al., 1990).

Considering the lower limit of G_Smax_ (0.15 mol H_2_O m^-2^ s^-1^d) for the optimum water status (Ramírez et al., 2016), stomatal limitation may have occurred in the present study, where the average value of G_Smax_ in the control at TI was 0.18 mol H_2_O m^-2^ s^-1^d (Figure 2). Nevertheless, the variation in A_max_ during TI was more closely associated with that in Φ_PSII_ (Table 4), indicating that non-stomatal limitation may have played a dominant role in restricting A_max_, which agrees with previous findings (Dwelle et al., 1981; Prange et al., 1990). Accelerated senescence by high temperature might reduce photosynthetic efficiency (Reynolds et al., 1990). However, photosynthesis was measured only on the young leaves in the present study. Moreover, HDT during TB did not reduce A_max_. These show that there are other limiting factors than accelerated senescence. Temperature above 38°C is likely to damage the PSII of potato leaves (Havaux, 1993). However, the PSII of potato leaves is known to acclimate under temperature slightly lower than 38°C, increasing the cardinal temperature by 5–8°C (Wolf et al., 1990; Havaux, 1993). This acclimation was processed through accumulation of zeaxanthin in the leaves, which increases membrane stability (Havaux and Gruszecki, 1993; Havaux et al., 1996). However, the temperature during TI was lower than 38°C. Moreover, the response of photosynthesis to high temperature was not instantaneous (Figure 2), which disagrees with the results of previous studies (Wolf et al., 1990; Havaux, 1993). These indicate that denaturation of PSII is not the reducing factor in the present study.

Recently, regulation of potato photosynthesis by source-sink relation at moderate-high temperature was reported (Hastilestari et al., 2018); moderate-high belowground temperature (29°C) with mild air temperature (22/20°C) causes decreased activity and expression of sucrose synthase (i.e., decreased tuber sink strength), depressed expression of the phloem-mobile tuberization signal SP6A in the leaves, and increased hexose in the leaves, thereby decreasing photosynthesis. In addition, Yan et al. (2011) found that Φ_PSII_ can be decreased by girdling, lowering sink demand from the tubers and roots in *Dahlia pinnata*. Hence, the decline in A_max_ caused by moderate-high temperature (31/18°C) at TI might be attributed to reduced tuber sink strength, as characterized by lowered Φ_PSII_. This feedback inhibition of photosynthesis can explain the slow response of photosynthesis to HDT (i.e., significant effect after a week) because the response of tuber development to environmental conditions is not instantaneous. Moreover, the negligible effect of HDT at TB on photosynthesis can be explained: TB is much less sensitive to high temperature than tuber development (Struik, 2007); thus, little or no feedback inhibition may occur at TB.

### Combined Effect of High Day and Night Temperatures

High day/night temperature during TI exhibited all the adverse effects of both HNT and HDT: decreased yield of large tuber, lowered harvest index, and reduced photosynthesis (Table 3 and Figure 2, 3). The extent of HDNT effect on the above traits was quite similar to that of the individual effects of HNT and HDT, and HDNT showed the most substantial yield loss. This result implies that the combined effect of HNT and HDT might be roughly additive owing to the different mechanisms of yield loss by HNT and HDT. Therefore, field studies regarding timing of warming will add substantial depth to the climate change study because daily temperature increases are diurnally asymmetric.

## Conclusion

The results of this study showed that the responses of photosynthesis and early tuber growth to high day and night temperatures in potato were quite different, although the response of early tuber yield was similar. During TI, HNT directly affected tuber development, resulting in a shift in tuber mass distribution and reducing early harvest index and early tuber yield. On the other hand, HDT lowered early tuber yield by restricting photosynthetic sources, which was probably determined by photosynthetic efficiency through a feedback inhibition. The combination of HNT and HDT exerted cumulative effects that resulted in the highest yield loss in HDNT. As tubers fully developed at TB, the thermal effects became negligible because most of the effects were directly or indirectly associated with tuber development.

Diurnal temperature range is widely believed to affect TI, growth, and yield of potatoes (Benoit et al., 1986; Bennett et al., 1991). In the present study, we could not reach any solid conclusion about the effect of diurnal fluctuation on those aspects. The effects of HNT and HDNT at TI on tuber mass distribution and harvest index were similar, indicating that the yield loss was caused by HNT, not by reduced DTR. This negligible effect of DTR may be attributed to the differences in sensitivity to DTR among varieties (Bennett et al., 1991), or it may occur because the DTRs at TI (>8°C) were within the optimum range. To isolate the effect of DTR from those of day and night temperatures, further field experiments using different varieties in different regions are needed.

## Author Contributions

Both authors conceived and designed the experiment and read and approved the final manuscript. Y-UK conducted the experiments, collected and analyzed the data, and wrote the manuscript. B-WL supervised the study and revised the manuscript.

## Conflict of Interest Statement

The authors declare that the research was conducted in the absence of any commercial or financial relationships that could be construed as a potential conflict of interest.

## Footnotes

1 http://www.fao.org/faostat/
